# Deep conditional generative model for personalization of 12-lead electrocardiograms and cardiovascular risk prediction

**DOI:** 10.3389/fdgth.2025.1558589

**Published:** 2025-04-16

**Authors:** Yuling Sang, Abhirup Banerjee, Marcel Beetz, Vicente Grau

**Affiliations:** ^1^ Centre for Computational Biology, Duke-NUS Medical School, Singapore, Singapore; ^2^ Institute of Biomedical Engineering, Department of Engineering Science, University of Oxford, Oxford, United Kingdom; ^3^ Division of Cardiovascular Medicine, Radcliffe Department of Medicine, University of Oxford, Oxford, United Kingdom

**Keywords:** cardiac MRI, cardiovascular disease, cardiovascular risk prediction, ECG electrodes, ECG generation, variational autoencoder

## Abstract

**Background:**

12-lead electrocardiograms (ECGs) are a cornerstone for diagnosing and monitoring cardiovascular diseases (CVDs). They play a key role in detecting abnormalities such as arrhythmias and myocardial infarction, enabling early intervention and risk stratification. However, traditional analysis relies heavily on manual interpretation, which is time-consuming and expertise-dependent. Moreover, existing machine learning models often lack personalization, as they fail to integrate subject-specific anatomical and demographic information. Advances in deep generative models offer an opportunity to overcome these challenges by synthesizing personalized ECGs and extracting clinically relevant features for improved risk assessment.

**Methods:**

We propose a conditional Variational Autoencoder (cVAE) framework to generate realistic, subject-specific 12-lead ECGs by incorporating demographic metadata, anatomical heart features, and ECG electrodes’ positions as conditioning factors. This allows for physiologically consistent and personalized ECG synthesis. Furthermore, we introduce a revised Cox proportional-hazards regression model that utilizes the latent embeddings learned by the cVAE to predict future CVD risk. This approach not only enhances the interpretability of ECG-derived risk factors but also demonstrates the potential of deep generative models in personalized cardiac assessment.

**Results:**

Our model is trained and validated on the UK Biobank dataset and *in silico* simulation data. By incorporating heart position and electrodes’ positions, the generated ECGs demonstrate strong consistency with *in silico* simulations, providing insights into the relationship between cardiac anatomy and ECG morphology. Furthermore, our CVD risk prediction model achieves a C-index of 0.65, indicating that ECG signals, together with demographic and anatomical information, contain valuable prognostic information for stratifying subjects based on future cardiovascular risk.

**Conclusion:**

This work marks a significant advancement in ECG analysis by providing a conditional VAE framework that not only improves ECG generation but also enriches our understanding of the relationship between ECG patterns and subject-specific information. Importantly, our approach enables clinically significant information to be extracted from 12-lead ECGs, providing valuable insights for predicting future CVD risks.

## 1 Introduction

The electrocardiogram (ECG) is a well established, non-invasive diagnostic tool that records the electrical activity of the heart over time (1). However, the manual analysis of ECG data can be a time-consuming and labor-intensive process, requiring significant expertise in interpreting complex patterns and abnormalities in the heart’s electrical activity. With the increasing use of wearable devices and other monitoring technologies, large volumes of ECG data can be generated on a daily basis (2), further exacerbating the challenge of manual analysis. As a result, there is a need for automatic techniques to facilitate the efficient diagnosis of heart diseases using the ECG.

Machine learning has emerged as a powerful tool for enabling automated analysis in a wide range of ECG-based tasks (3–9). While machine learning techniques have shown great promise, many of these methods require large amounts of labeled data to effectively train the model. This poses a significant challenge as obtaining and annotating large datasets can be time-consuming, expensive, and resource-intensive. Also, class imbalance is another common issue in ECG datasets, as certain cardiac abnormalities may be relatively rare compared to normal ECG patterns, which can lead to biased model performance (10). Furthermore, preserving patient privacy is another critical aspect of medical data sharing and usage, especially in the context of ECG data, which may contain personally identifiable and sensitive health information (11).

Researchers have tried to solve these problems through data augmentation. Classic data augmentation methods such as performing translation and adding noise can only obtain limited new additional information, which may lead to overfitting during the training process. In order to truly augment the dataset, deep generative models have attracted attention in recent years for the generation of high-quality synthetic medical data, and been applied successfully in ECG research. Previous deep generative models (12–14) have mainly focused on only single-lead ECG generation and lack the introduction of subject characteristics. 12-lead ECGs are the clinical gold standard, providing comprehensive spatial information about cardiac conduction, and incorporation of demographic and physiological features is crucial for understanding the relationship between ECG morphology and subject information. The inability to generate physiologically consistent multi-lead signals significantly restricts the applicability of these models in personalized cardiac assessments, as key inter-lead relationships and subject-specific variations are not considered.

Traditional simulation methods, such as the Extracellular-Membrane-Intracellular (EMI) model or the work of Mincholé et al. (15), which utilized computer simulation with torso-ventricular anatomical models to investigate the impacts of ventricular and torso anatomy on 12-lead ECGs, hypothesize that geometrical factors, including ventricular anatomy, heart orientation, location, and torso anatomy, differentially influence QRS complexes in 12-lead ECGs. Although these traditional biophysically-based models can be very precise, they are computationally intensive, with simulations requiring up to several hours (16), whereas generative models can synthesize ECG signals in milliseconds per sample.

Our study aims to bridge these gaps by introducing a conditional Variational Autoencoder (cVAE) framework that generates 12-lead ECGs conditioned on anatomical features. In our previous work (17), we included subject metadata and anatomical characteristics, such as heart positions and orientations, from cardiac Magnetic Resonance Imaging (MRI) to develop a cVAE model that can generate realistic 12-lead ECGs with ability to capture useful features from different conditions. However, the generated conditional ECGs only partially align with the *in silico* data, likely due to the absence of torso structural information in the model.

To address this limitation, in this study, we incorporate ECG electrode locations as additional input features. A widely used configuration for ECG measurement involves 10 electrodes: 4 electrodes placed on the limbs [left arm (LA), right arm (RA), left leg (LL), and right leg (RL)] and 6 electrodes positioned on the chest (V1 to V6). These chest electrodes provide detailed spatial information about the heart’s electrical activity, enabling the formation of 12 leads and establishing a strong connection between the torso structure and ECG signals. With the recent development of automated 3D torso reconstruction (18, 19), we are able to obtain the precise electrodes’ positions from each subject’s clinical MRI. This additional information provides valuable constraints to the model, allowing it to generate ECGs that are not only realistic but also anatomically and physiologically consistent.

In order to demonstrate the efficacy of the latent representation achieved from the VAE architecture, we extend the model to perform future cardiovascular disease (CVD) risk prediction. The majority of contemporary algorithms focusing on CVD risk prediction are based on a limited set of subject attributes, e.g., age, smoking history, and blood pressure. Recently, efforts have been made to investigate a broader range of risk predictors, encompassing interaction terms and employing more sophisticated machine learning techniques to model CVD risk (20). However, these studies have only considered tabular data, neglecting other potential information sources such as ECG or MRI. Recent studies (21–23) have increasingly shown that ECG abnormalities are a promising predictor of CVD risk, making the direct use of ECG signals an attractive direction for risk stratification. However, most previous approaches have relied solely on ECG data without incorporating the underlying anatomical context. Specifically, variations in heart position and orientation can substantially alter ECG morphology by shifting the electrical axis and modifying the amplitude and duration of key waveforms (15, 19). If these anatomical effects are not accounted for, normal variations in heart position may be misinterpreted as pathological changes or, conversely, true abnormalities might be obscured. By incorporating heart position and orientation, our model can disentangle these anatomical influences from disease-related signals. Therefore, our work explores the novel integration of heart data with ECG signals, aiming not only to generate more realistic ECGs but also to enhance the accuracy of CVD risk prediction by incorporating critical anatomical context.

Our study makes the following key contributions:

1. We develop a novel cVAE framework capable of generating 12-lead ECGs and incorporate patient-specific conditions.
2. We demonstrate that incorporating heart position and electrodes placement significantly improves the fidelity of synthetic ECG signals, capturing inter-lead dependencies and individual variability.
3. We introduce a revised Cox proportional-hazards model, leveraging ECG-derived latent embeddings to enhance CVD risk prediction.
4. ECG signals, combined with anatomical context, can stratify subjects based on their future cardiovascular risk (C-index = 0.65), providing valuable insights for personalized cardiac assessments.

## 2 Materials and methods

### 2.1 ECG dataset

Our research has been conducted using the UK Biobank Resource under Application Number “40161” (24). In total, we have ECG files from 37,508 volunteers, together with their personal information including age, sex, BMI, and their clinical imaging information.

Each ECG file in the UK Biobank dataset contains a 10-s sample recorded at 500 Hz with 5,000 data points per lead. Additionally, UK Biobank provides a median beat waveform, which is computed by extracting individual heartbeats from the 10-s segment, aligning them, and calculating the median waveform across all beats. This median beat contains approximately 600 data points and serves as a representative single heartbeat, The majority of our experiments are performed on the shorter median data, since the averaging process can help to reduce noise and artifacts in the signal, providing a cleaner and accurate representation of the cardiac activity. It not only allows us to focus on specific features of the ECG, such as the QRS complex, without the confounding effects of beats variability in the longer recording, but requires less computational power and time as well. The ECG data require some additional pre-processing to remove artifacts like baseline drift, which was removed using a finite impulse response band-pass filter between 3–45 Hz, inspired by an entry to the Computing in Cardiology (CinC) 2017 challenge (25).

The age and sex information of the subjects are included in the UK Biobank ECG files. The BMI can be located within the “Body Size Measures” category in the UK Biobank, accessible through each subject’s unique identification number.

The UK Biobank dataset we use includes 21,083 cardiac MRI cases in total, and they were acquired at the same date as the ECG acquisitions (26). These cardiac MRI are used to calculate subject-specific information, including heart positions, orientations and electrode positions.

### 2.2 CVD risk prediction dataset

**Figure 1:**
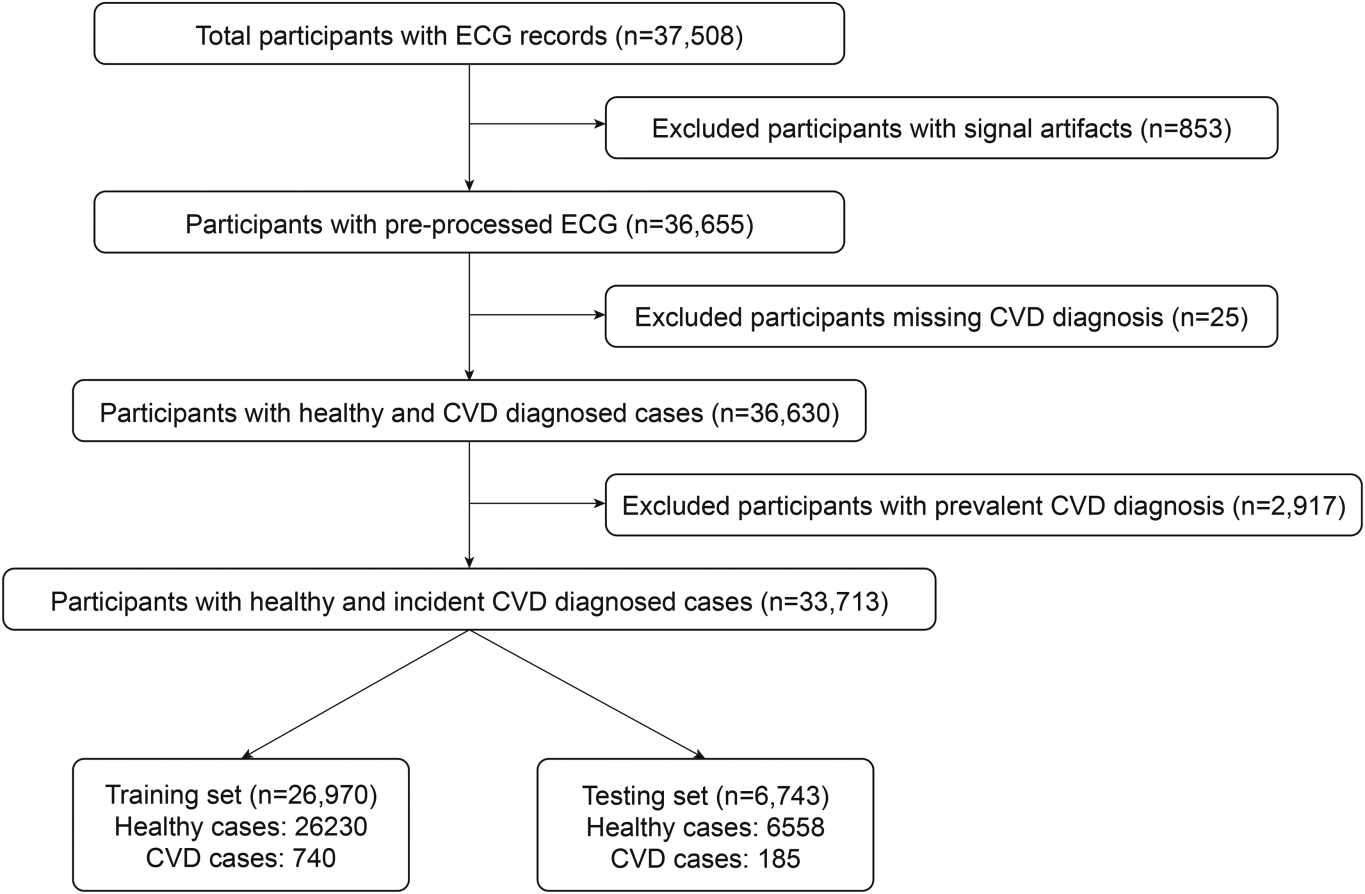
CVD risk prediction dataset preparation diagram.

In this project, we define CVD as a composite of any of the following ICD-10 diagnosis codes: I20 (angina pectoris), I21 (acute myocardial infarction), I22 (subsequent myocardial infarction), I23 (certain current complications following acute myocardial infarction), I24 (other acute ischaemic heart diseases), I25 (chronic ischaemic heart disease), and I50 (heart failure). This is similar to the research of Alaa et al. (20), but we exclude I60–I69 (cerebrovascular diseases), as we assume that the link between ECG and cerebrovascular disease is relatively weak. We also exclude vascular dementia, since at the time of our study we do not have access to its ICD-10 code. We apply our model only on the cases whose CVD event date is posterior to the ECG acquisition date, which we refer as incident cases. We identify all subjects for which a CVD event was recorded before ECG acquisition as prevalent CVD cases (27). The diagram of our dataset preparation is shown in Figure 1.

In total, we have 37,508 subjects with successful ECG recordings. As detailed in Section 2.1, a finite impulse response band-pass filter is applied to correct baseline drift in the signals. However, this method does not address short peak artifacts, which can significantly affect our model training. To mitigate this issue, we remove all signals with absolute amplitudes exceeding 800 mV/100 in any lead, resulting in the exclusion of 853 subjects. A further 25 subjects are excluded due to missing CVD diagnoses. Next, we exclude 2,917 subjects with prevalent CVD diagnosis from our dataset leaving 33,713 subjects. Among them, we separately have 925 cases with incident CVD diagnosis and 32,788 healthy subjects with no CVD records at the time of this study.

We allocate 80% of each healthy and CVD group into the training set and the remaining 20% into the test set for CVD risk prediction. This stratification was applied separately to each of the 7 CVD subtypes, ensuring that their proportions remained consistent across both sets. By maintaining balanced representation, we reduce the potential for certain diseases to be over- or under-represented, thereby improving model accuracy and generalizability.

### 2.3 Heart position and orientation

**Figure 2:**
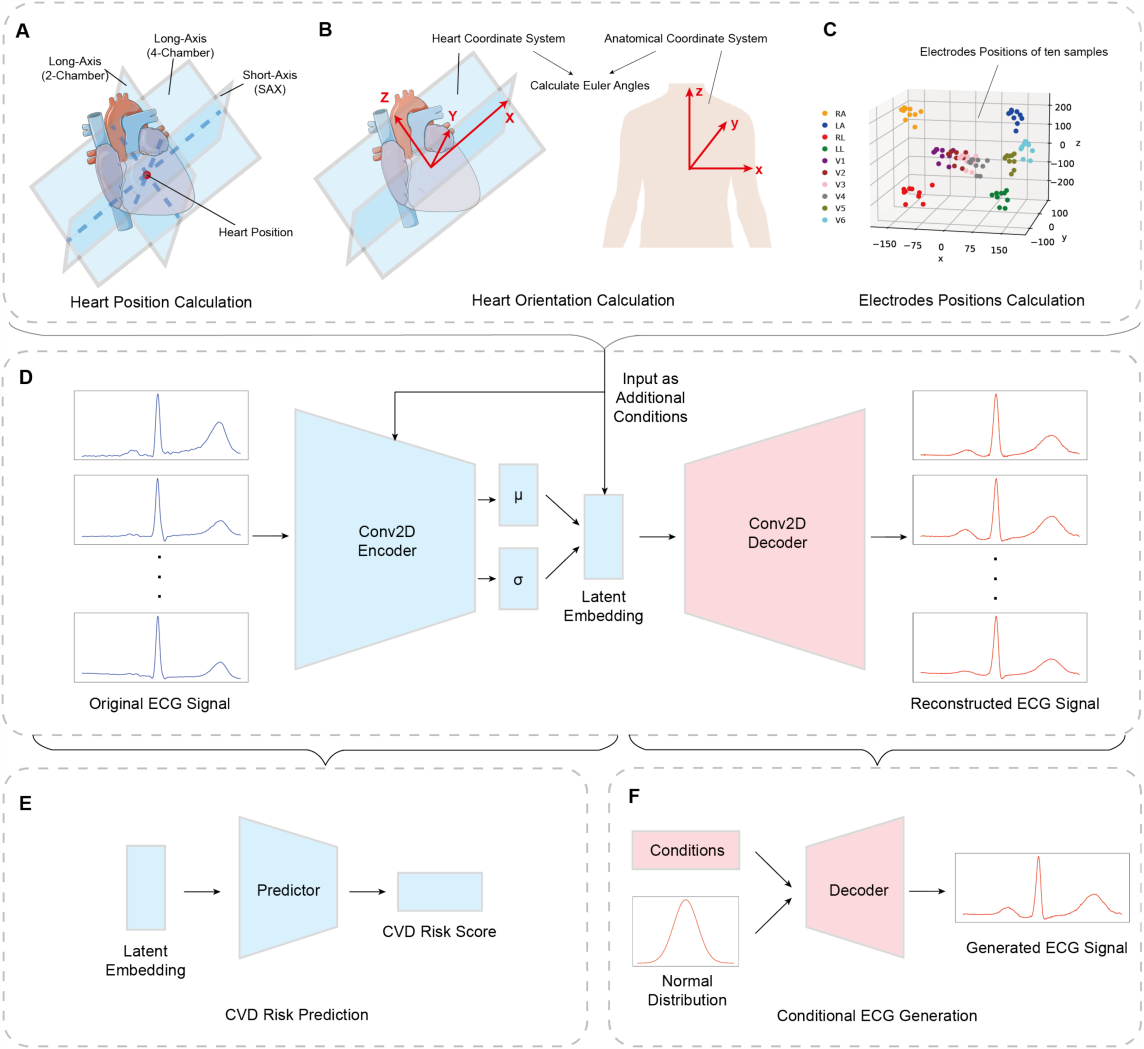
Overall pipeline for utilizing heart and torso information in conditional ECG generation and CVD risk prediction. **(A)** Heart position is calculated by the intersection of 2-chamber view, 4-chamber view, and middle short-axis view. **(B)** Heart orientation is represented by Euler angles between heart coordinate system and anatomical coordinate system. **(C)** Electrodes’ positions are achieved from cardiac MRI (18) and transformed to heart coordinate system. **(D)** The conditional VAE architecture with the heart and torso information as additional condition inputs added to the first fully-connected layer of encoder and latent space. **(E)** The CVD risk prediction model. An additional predictor is concatenated to the latent embedding, which provides the risk score to realize the revised Cox proportional hazard regression model. **(F)** The conditional ECG generation is performed by trained decoder, which takes random sampling from normal distribution and condition inputs.

The heart position and orientation data are calculated using information from the cardiac MRI. In general, a standard cardiac MRI acquisition includes a stack of 2D short-axis (SAX) slices, which cover the left and right ventricles from apex to base, as well as a 2-chamber long axis (LAX) slice and a 4-chamber LAX slice (28). As shown in Figure 2A, we define the heart position as the intersection between three planes: 2-chamber LAX plane, 4-chamber LAX plane, and the middle plane of the SAX view stack. The definition of a plane is 3D space is given by Equation 1:

(1) n⋅(X−P)=0

where:

• $n\in R^{3}$ is the normal vector of the plane;
• $X=(x,y,z)\in R^{3}$ is an arbitrary point on the plane; and
• $P=(P_{x},P_{y},P_{z})$ is a known point on the plane, extracted from the DICOM metadata.

The specific plane equations for the three anatomical planes are shown in Equations 2–4:

(2) $$n_{\text{SAX}}\cdot (X-P^{\text{SAX}})=0$$

(3) $$n_{\text{2CH}}\cdot (X-P^{\text{2CH}})=0$$

(4) $$n_{\text{4CH}}\cdot (X-P^{\text{4CH}})=0$$

where $n_{\text{SAX}},n_{\text{2CH}},n_{\text{4CH}}$ are the normal vectors of the SAX, 2-chamber LAX, and 4-chamber LAX planes, respectively. $P^{\text{SAX}},P^{\text{2CH}},P^{\text{4CH}}$ are the image position points for each plane. By solving this system of three linear equations, we obtain the heart's center position, as shown in Equation 5:

(5) $$P_{\text{heart}}=(x_{h},y_{h},z_{h})=\text{Intersection}(\text{SAX, 2CH, 4CH})$$

The heart orientation is defined relative to the standard anatomical coordinate system using a new heart-specific coordinate system based on the SAX and 4-chamber LAX planes. This coordinate system is denoted as $\left(e_{X},e_{Z},e_{Y}\right)$. The new X-axis is computed as the normalized intersection vector between the SAX and 4-chamber LAX planes:

(6) $$e_{X}=\frac{L_{\text{SAX-4CH}}}{\left\|L_{\text{SAX-4CH}}\right\|}$$

where $L_{\text{SAX-4CH}}=n_{\text{SAX}}\times n_{\text{4CH}}$ is the direction vector of the line formed by the intersection of the SAX and 4-chamber LAX planes. $\left\|L_{\text{SAX-4CH}}\right\|$ is the vector norm, ensuring $e_{X}$ is a unit vector.

The new Z-axis is chosen to be perpendicular to the 4-chamber LAX plane, while ensuring it remains orthogonal to $e_{X}$:

(7) $$e_{Z}=n_{\text{4CH}}-(n_{\text{4CH}}\cdot e_{X})e_{X}$$

where $n_{\text{4CH}}$ is the normal vector of the 4-chamber LAX plane. The term $(n_{\text{4CH}}\cdot e_{X})e_{X}$ removes the component of $n_{\text{4CH}}$ that is parallel to $e_{X}$, ensuring orthogonality.

The new Y-axis is computed as the cross-product of $e_{X}$ and $e_{Z}$:

(8) $$e_{Y}=e_{X}\times e_{Z}$$

Equations 6–8 ensure that $\left(e_{X},e_{Y},e_{Z}\right)$ forms a right-handed orthonormal coordinate system. The Euler angles describe the rotation between the heart coordinate system ($e_{X},e_{Y},e_{Z}$) and the standard anatomical coordinate system (x,y,z), as described in Equations 9–11:

(9) $$\alpha =\cos^{-1}\;\left(\frac{e_{X}\cdot x}{\left\|e_{X}\|\cdot \|x\right\|}\right)$$

(10) $$\beta =-\cos^{-1}\;\left(\frac{e_{Z}\cdot z}{\left\|e_{Z}\|\cdot \|z\right\|}\right)$$

(11) $$\gamma =-\cos^{-1}\;\left(\frac{e_{X}\cdot N}{\left\|e_{X}\|\cdot \|N\right\|}\right)$$

where N is the normal vector of the anatomical XOY plane.

### 2.4 Electrode positions

We use the work of Smith et al. (19) for estimating the electrodes’ positions for each subject. The method applies a U-net deep learning network for automated torso segmentation and contour extraction from the localizer and scout cardiac MRI from the UK Biobank dataset (18). The undesired section including head, neck, and arms and potential artifacts such as shadow regions are removed using a preprocessing algorithm. Finally, a statistical shape model is used over sparse 3D contours to generate 3D torso meshes, with the electrodes’ positions estimated on the 3D torso meshes.

Due to the relative slow speed of this algorithm, which usually takes 30–60 min for one case, we reconstruct a total of 1,834 3D torso meshes, and measure the ten electrodes’ positions for standard 12-lead ECGs, which include four limb electrodes including left arm, right arm, left leg, and right leg, and six precordial electrodes corresponding to six precordial leads. Figure 2C presents electrodes’ locations of ten sample cases from our training set.

The electrodes generated from torso meshes are 3D variables located in the anatomical coordinate system presented in Figure 2B. In order for each subject’s location information to be more accurately comparable and representative of the anatomical characteristics of the heart, we utilize the heart position and orientation calculated before, to transfer the locations from anatomical coordinate system to heart coordinate system. In this way we capture the corresponding relationship between the electrode coordinates and the heart coordinates while treating the heart coordinates as the origin. Therefore, the electrodes’ positions information is able to contain both torso and heart features.

### 2.5 Conditional VAE architecture

Assuming that the original data set is x, the encoder produces a hidden variable z and the decoder produces the reconstructed dataset $\hat{x}$. The VAE aims to learn the marginal likelihood of the input through this generative process, as defined in Equation 12:

(12) $$\max_{\phi ,\theta }E_{q_{\phi }(z|x)}[\log\;p_{\theta }(x|z)]$$

where ϕ, θ parameterize the distributions of the VAE encoder and decoder respectively. Here, $q_{\phi }(z|x)$ is the approximate posterior distribution of the latent variable z given the input x, and $p_{\theta }(x|z)$ represents the likelihood of the input given the latent variable, modeled by the decoder. Based on the evidence lower bound (ELBO), the training process of VAE uses the loss function as Equation 13:

(13) $$L=-E[\log\;p_{\theta }(x|z)]+D_{KL}(q_{\phi }(z|x)\|p(z))$$

where $-E[\log\;p_{\theta }(x|z)]$ in our experiment is chosen as the mean-squared error between the original **x** and the reconstructed $\hat{x}$, denoted as $L_{\mathrm{recons}}$. $D_{KL}(q_{\phi }(z|x)\|p(z))$ represents the Kullback-Leibler (KL) divergence between predefined posterior p(z)∼N(μ,σ) and the latent space distribution $q_{\phi }(z|x)∼N(\mu_{z},\sigma_{z})$ produced by our network, denoted as $L_{KL}$. The posterior p(z) is set as a standard normal distribution for easy computation.

The structure of the cVAE is similar to VAE, except that category information y is added as part of the input data, which is used to control sample generation for specified categories. The modified objective function of cVAE is presented in Equation 14:

(14) $$L=-E[\log\;p_{\theta }(x|z,y)]+D_{KL}(q_{\phi }(z|x,y)\|p(z))$$

Figure 2D shows the revised cVAE network architecture. In the encoder part, the ECG data, with the dimension of 1×12×400, is treated as the input, followed by two convolutional blocks, each of which includes a 2-dimensional convolution layer, a batch normalization layer, and an Exponential Linear Unit (ELU) activation function. Next, we have an Average Pooling layer and the output is flattened. We use two fully-connected layers to produce two 64-dimensional vectors: one is interpreted as the mean, while the other one is considered as the logarithms of the variance of 64 normal distributions. In the final stage, a sampling layer is used to get a 64-dimensional latent space sampled from the distributions mentioned above. The decoder part is symmetrical to the encoder part, which uses upsampling layers and 2-dimensional deconvolution layers, to reconstruct the 12 lead ECGs.

Physiologically, heart position, orientation, and electrode locations define the spatial relationship between the heart’s electrical activity and the recording leads, thereby affecting ECG waveform morphology. To ensure that the generative model learns these dependencies, in the encoder, the conditional information is concatenated to the first fully connected layer, ensuring that the learned latent representation z captures the variability introduced by anatomical differences. In the decoder, the same conditional inputs are incorporated alongside z to modulate ECG generation, enforcing physiological consistency by reconstructing ECG waveforms that align with the given heart position, orientation, and electrode locations. Its dimension c depends on the information category: for heart position and orientation, these are three-dimensional coordinates and angles respectively, and for electrodes’ positions are 10×3 dimensional coordinates.

The model is implemeted in Python3 using PyTorch. Adam optimizer (29) is used with a learning rate of 0.001. For each VAE, we assigned 80% of the dataset as the training set and the rest as test set. The batch size is set as 64, and the training process is performed for 80 epochs. We run all experiments on NVIDIA A100 Tensor Core GPU.

### 2.6 Risk prediction model architecture

The revised network presented in Figure 2E is the addition of an extra predictor connected to the latent space so that we are able to analyze the representations and features contained in latent space and achieve a risk score output. For the predictor, we perform all experiments on a single fully connected layer, with 64 latent space dimension as input, and one dimensional risk score as output, obtained using a sigmoid function.

The loss function of this predictor network consists of three parts. The first two parts are the same as previous sections, i.e., $L_{KL}$ and $L_{\mathrm{recons}}$, as shown in Equations 13 and 14. We use the Cox proportional hazard regression model to realize the last survival loss part. Typically in a linear Cox model, the hazard function has the form defined in Equation 15:

(15) $$h(t,x_{1},\ldots ,x_{m},\beta_{1},\ldots ,\beta_{m})=h_{0}(t)\exp\;\{\beta_{1}x_{1}+\cdots +\beta_{m}x_{m}\}$$

where $h_{0}(t)$ is the baseline hazard function, which would correspond to a hypothetical subject whose covariate values are all zeros. The $\exp\;\{\beta_{1}x_{1}+\cdots +\beta_{m}x_{m}\}$ is called the relative risk of a subject. Predictor covariate variables, $x_{i}$ , are weighted by $\beta_{i}$, to adjust this baseline hazard function for each subject. These weights, $\beta^{\prime }$, are estimated by maximising the Cox proportional hazards partial likelihood function:

(16) $$\log\;L(\beta )=\sum_{i=0}^{n}\delta_{i}\left(\beta^{\prime }x_{i}-\log\;\sum_{\;j\in R(t_{i})}e^{\beta^{\prime }x_{j}}\right)$$

where $x_{i}$ is the vector of predictor covariate variables, $\delta_{i}$ is a boolean variable indicating event status, and $R(t_{i})$ is the set of subjects yet to have an event or be censored at time t for subject i. Equation 16 can be adapted for a neural network by replacing $\beta^{\prime }x_{i}$ with the output of a network.

Therefore, in order to optimize our VAE network training for survival analysis, we replace $\beta^{\prime }x_{i}$ with the output of our predictor, as shown in Equation 17, to form our survival loss function:

(17) $$L_{\mathrm{surv}}=\frac{1}{N}\sum_{i=0}^{N}\delta_{i}\left(r_{i}-\log\;\sum_{\;j\in R(t_{i})}e^{r_{\;j}}\right)$$

where N is our batch size and $r_{i}$ is the sigmoid of the output of the model, i.e., the log-hazard ratio of subject i. Preliminary work with the survival model showed that the exponent term in $L_{\mathrm{surv}}$ can cause the untrained predictor head to exponentiate large numbers leading to numerical instability. To prevent this, we apply a sigmoid function to the output of the model, both ensuring that large exponents are not possible and keeping the relative order of risk for subjects unchanged, since the sigmoid is monotonically increasing.

Therefore, the loss for our overall model is written as:

(18) $$L_{\mathrm{total}}=L_{\mathrm{recons}}+L_{KL}+L_{\mathrm{surv}}.$$

As this study represents an initial investigation, the three loss terms in Equation 18 are assigned equal weights. In the future, techniques such as grid search or other hyperparameter optimization methods can be utilized to systematically determine the optimal weight configuration.

## 3 Results

### 3.1 ECG conditioned on heart position and orientation

We used all 21,083 UK Biobank cases that include both ECG and MRI data to train our model. After training, by modifying the conditioning inputs (i.e., heart position and orientation), we generated synthetic ECGs reflecting various cardiac poses, which we then compared with the simulation trends reported by Mincholé et al. The conditional ECG generation is performed by the pre-trained decoder which takes random samples from a normal distribution and conditional inputs, shown in Figure 2F.

**Figure 3:**
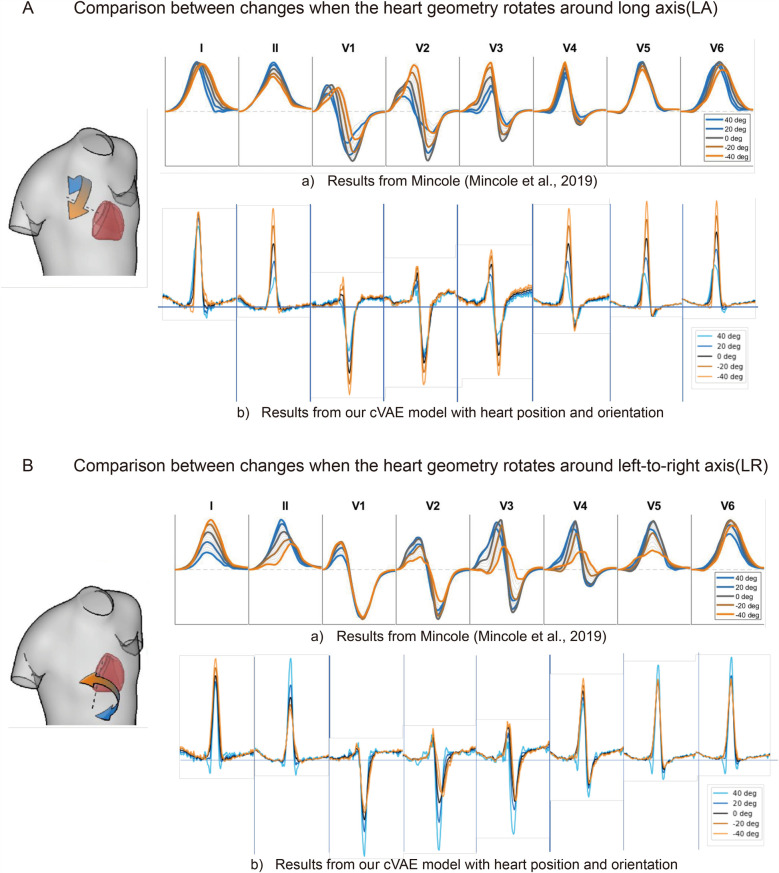
The comparison of ECG changes between previous work (15) and proposed cVAE model in leads I, II, and V1 to V6 when heart rotates around long axis **(A)** and around left-to-right axis **(B)** in 40, 20, 0, −20, 40° separately.

**Figure 4:**
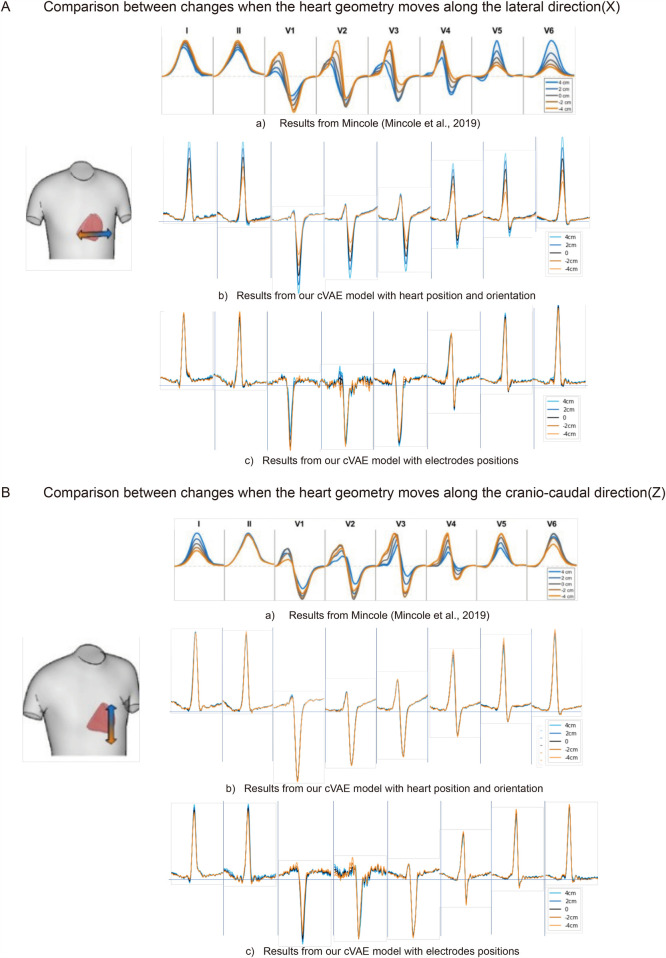
The comparison of ECG changes between previous work (15) and proposed cVAE model in leads I, II, and V1 to V6 when heart moves along the lateral direction **(A)** and along the cranio-caudal direction **(B)** in 4, 2, 0, −2, −4 cm separately.

Figures 3 and 4 show the results that reflect the learned effect of heart rotation and translation, respectively. For rotation, we first rotate the heart along the long axis, which is the Z axis of the heart coordinate system, and then left-to-right ventricle axis, which is the Y axis of the heart coordinate system. For translation, we move the heart along the lateral and cranio-caudal directions, which would be represented as the heart position coordinate (x,y,z) changes, so that moving along the lateral direction and cranio-caudal direction means changing the value of x and z respectively.

We compare our generated ECGs with the work of Mincholé et al. (15). As shown in Figure 3A(b), rotation along the long axis influences R, S, and T waves in almost all the ECG leads. The heart rotated more counterclockwise results in an increase in the amplitude of these waves. After comparison, we find only lead V4 completely agrees with the result of Mincholé et al. (15) [Figure 3A(a)], while leads V1 to V3 have the same change on R wave but the opposite on S wave. The rest of the leads show different features, for in results of Mincholé et al. (15) long axis rotation exerts a limited influence on leads I, V5, and V6.

Figure 3B(b) shows the amplitude of R and S waves increase in lead II and V1–V3 when we rotate more counterclockwise along the left-to-right ventricular axis. More clockwise rotation affects the morphology of S wave in leads V2 and V3. Five leads I, II, V2, V3, and V5 in our work share the same amplitude features with results of Mincholé et al. (15). Our results also reflect the influence on the morphology, but the degree of change is not as prominent as Figure 3B(a).

In Figure 4A(b), when the heart moves more to the left-hand direction, the R wave and T wave amplitudes increase in leads I, II and V4-V6, while the S wave amplitude increases in all precordial leads. After comparison, we find only leads V5 and V6 agree with the findings of Mincholé et al. (15) [Figure 4A(a)], while other leads reflect the opposite influence.

Finally, we analyze the translation along the cranio-caudal direction. Figure 4B(b) shows that translation along this direction mainly affects the amplitude of T wave of leads V2-V4. We also notice an increase after translating the heart more to the inferior direction in leads V4-V6. Compared to the work of Mincholé et al. (15) [Figure 4B(a)], only lead II completely agrees. While our V4 and V5 have similar response to this translation, the degree of change in work of Mincholé et al. (15) are much greater.

### 3.2 ECG conditioned by electrode positions

While the previous results demonstrate that our network successfully extracts valuable and relevant features from the ECGs, incorporating only heart position and orientation may present certain limitations. For instance, only considering absolute heart coordinates without accounting for their relative positions in the torso structure may reduce comparability across subjects, as the anatomical coordinate origin is determined by the scanner. This highlights the potential benefits of incorporating additional factors, such as torso structure, to enhance the accuracy and generalizability of our approach. We include the ten electrode positions to fix our torso structure when we perform the heart position translation. Each electrode position is transformed from anatomical coordinate system to heart coordinate system using heart position and orientation Euler angles. Therefore, when evaluating the influence of heart information, the electrodes’ positions are the only condition inputs to the model, which contain both heart position, orientation, and torso information.

For the analysis Figures 4A(c),B(c), we use a subset of 1,834 real cases that include electrode position information. By fixing the electrode positions to control for torso influence and modifying heart position inputs, we generate ECGs that are compared with the morphological trends observed in Mincholé’s work. From Figure 4A, in general, the generated ECGs using ten electrodes have the same quality as the results using only heart positions, except with more noise in the generated leads V1 and V2 signals. This increased noise may result from the mismatch between the 3D nature of electrode positions and the 1D latent space used in our model, which introduces additional complexity in the decoding process.

The overall impact of heart information on the generated signals are more obvious than the one using electrodes, with more clear difference when we move the heart. However, if we treat the simulated signals in Figure 4A(a) as the standard, we can discover more accurate features or trends presented in the electrodes based model. When we look at leads I and II, Figure 4A(b,c) reacts to the position change in a completely opposite way. While R peak amplitude increases with the right to left movement of the heart in Figure 4A(b), it decreases in Figure 4A(c). When it comes to precordial leads, in leads V1 and V4 our electrodes’ results of Figure 4A(c) also have more consistency with the simulated results than ones with heart-position only [Figure 4A(b)]. When the heart moves more to the left, the S wave peak of lead V1 increases, while in lead V4, the R wave peak increases and the S wave peak decreases. Those characteristics are exhibited in the opposite direction in Figure 4A(b).

In Figure 4B, more noise can be found in leads V1 and V2 in the model with electrodes’ positions. Compared to Figure 4B(b), the influence of Z direction change is revealed more clearly using electrodes. Especially in leads I and II of the model with electrodes’ positions [Figure 4B(c)], when the heart moves towards the head direction, the R wave amplitude will get increased, which is also reflected by the simulated results in Figure 4B(a). As comparison, the heart movement in Z direction has little influence on the final generated signals in our model with heart information only [Figure 4B(b)]. Regarding the precordial leads V1–V6, our two networks in Figures 4B(b),B(c) do not reveal large differences about the reaction to the heart position change. In leads V2–V6, the R and S wave amplitudes get larger if we move the heart more towards the feet. In general, the features in both Figures 4B(b),B(c) demonstrate more consistent results with simulated results in Figure 4B(a), except lead V6 which shows the opposite.

### 3.3 CVD risk prediction

**Figure 5:**
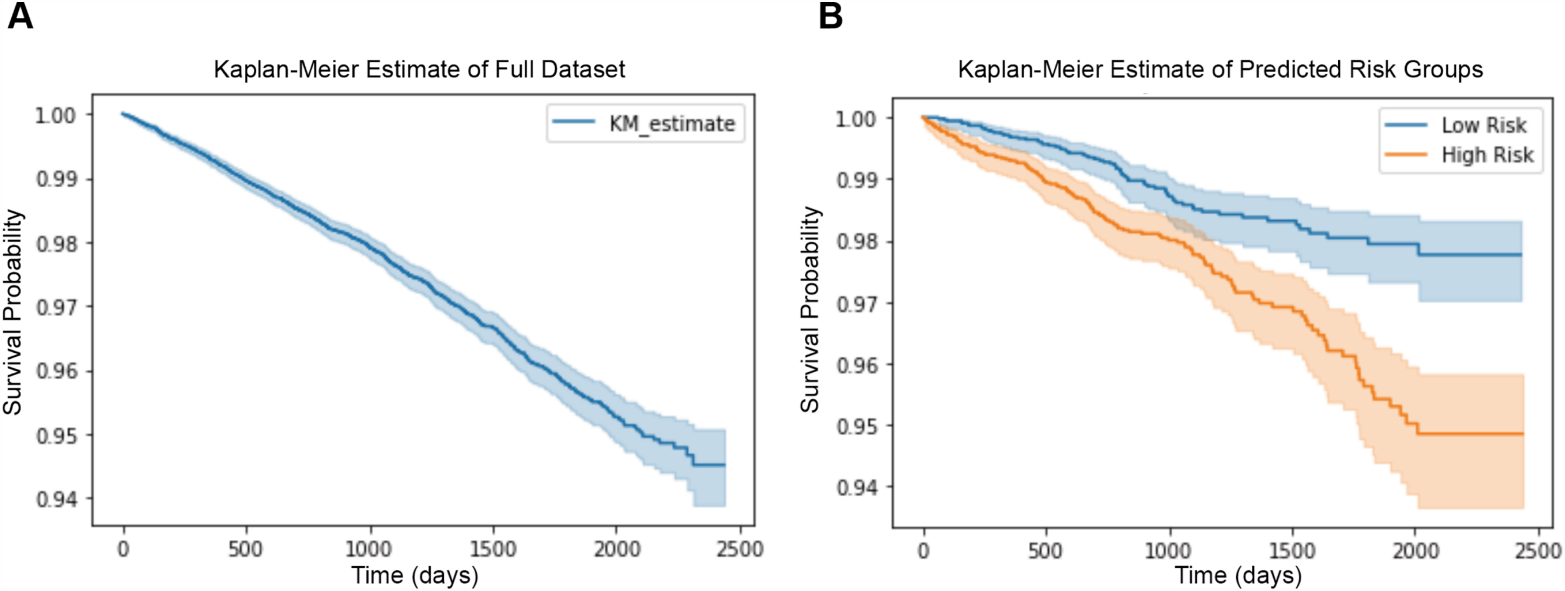
**(A)** Kaplan–Meier plot of the full dataset before stratification, showing survival probabilities for all subjects. **(B)** Kaplan–Meier plot of the test set, divided into low-risk (blue line) and high-risk (orange line) groups based on risk scores predicted by our model. The shaded areas represent the 95% confidence intervals (CIs).

We plot the Kaplan–Meier estimate curve of the full dataset before any stratification, as shown in Figure 5A. During seven years of follow-up observation, 5.5% of our total subjects have been diagnosed with CVD.

We train our network and achieve the score for each subject’s future CVD risk in our test set, and accordingly divide them into two groups: low CVD risk and high CVD risk, using the median risk as threshold. Figure 5B reveals the Kaplan–Meier estimate of both groups of our test set. In Figure 5B, we can notice a clear difference between low CVD risk and high CVD risk groups. The CVD event occurs in 2% of subjects in the low risk group, and 6% of subjects in the high risk group over a nearly 7-years observation period.

Instead of considering the absolute survival times for each occurrence, survival analysis frequently uses the relative risk of an event (30, 31). To evaluate this, we use the concordance index (C-index), a widely used metric in survival analysis. Unlike classification metrics such as AUC-ROC, sensitivity, and specificity, which require binary labels, the C-index assesses how well the predicted risk scores preserve the correct ranking of event times. This makes it particularly suitable for our task, where the goal is quantifying relative CVD risk rather than classifying individuals into discrete risk categories.

Our model achieves a C-index of 0.63, indicating that ECG-derived risk scores successfully rank individuals based on their future CVD risk with performance significantly above random chance (C-index = 0.5). While existing CVD risk models often achieve higher C-index values by incorporating comprehensive clinical and lifestyle factors (e.g., blood pressure, cholesterol, and smoking history), our study focuses specifically on evaluating the prognostic value of ECG morphology alone.

We also explore whether the additional information can improve the performance of the network. Therefore, we first introduce the sex and age to the encoder and next the electrodes’ positions. The idea was that sex and age are directly predictive of incident CVD, while the electrodes’ positions could be used by the network to contextualize the shape of the ECG and refine the prediction. For the prediction model including sex and age, we use the same training set and test set as in the previous sections. The first row of Table 1 shows the result of our baseline model with C-index of 0.63. The inclusion of heart position information resulted in an increase of 3% in the concordance index, indicating an improvement in the model’s ability to correctly rank individuals by CVD risk.

**Table 1:** C-index result for ECG baseline prediction model and model with additional demographic information.

Model	C-index
Baseline	0.63
Baseline + sex + age	0.65

For the model including electrodes’ positions, due to the limited size of our processed dataset as discussed in Section 2.4, we include 1,600 healthy cases and 100 cases with CVD diagnosed and maintain the same group proportion as in the previous experiment. From the results presented in Table 2, we find that the baseline model only achieves the C-index of 0.58. The addition of sex and age information increases the baseline model result to 0.61, with a 5% improvement. By incorporating electrodes’ positions relative to heart coordinate system, the revised model provides a 1.7% increase in C-index, from 0.58 to 0.59. However, including the electrodes’ position along with sex and age information do not further improve the predictive performance.

**Table 2:** C-index result for ECG baseline model, model with demographic information, and electrodes’ positions.

Model	C-index
Baseline	0.58
Baseline + electrode positions	0.59
Baseline + sex + age	0.61
Baseline + sex + age + electrode positions	0.61

## 4 Discussions

In this work, we have developed a conditional VAE model to generate 12-lead ECGs, which takes heart position, orientation, and electrodes’ positions as conditions. The results of our cVAE model show that the heart position and orientation have a significant impact on the generated ECGs, which is consistent with previous research (15). However, the influence of heart position and orientation on the generated ECGs is not as prominent as the simulated results. One possible explanation is that our position definition is not accurate enough because we only calculate the intersection of three cardiac MRI planes. An alternative explanation is in the work of Mincholé et al. (15) the torso structure was fixed for simulation, while in our research the torso of each subject can vary. Additionally, after comparison we find that some rotation degree and translation distance in the work of Mincholé et al. (15) are too large to the extent that they do not occur in real subjects.

When we include electrodes’ positions as input, they should also contain heart position and orientation information. During training, electrode positions help capture the influence of torso anatomy on ECG morphology. During generation, fixing electrodes’ positions allows us to control for torso-related variability, ensuring that observed ECG changes are primarily driven by modifications in heart position and orientation. Therefore, in this experiment we are able to reduce the influence of torso on our final generated results. From Figure 4, we can notice with the addition of electrodes’ positions, the consistency between our generated signals and simulated *in silico* signals of Mincholé et al. (15) gets improved. This illustrates that our model including the electrodes’ positions is capable of capturing useful features that represent the individual characteristics well, though there is more noise in the final generated signals. A potential explanation for this issue lies in the difference between the 3D nature of the electrodes’ positions (3×10 coordinates) and the 1D latent space (64 dimensions) used in our experiments. This mismatch introduces additional complexity, which may challenge the decoder’s ability to effectively interpret and reconstruct the information. To address this, further parameter tuning or introduction of a separate encoder for electrodes’ positions could help achieve better results.

While comparing our generated outcomes with the work of Mincholé et al. (15), it is important to acknowledge that the comparison is largely qualitative in nature, given that their work does not provide actual values to enable a more comprehensive, quantitative comparison. Thus, although this comparison provides some initial insights into the relative performance of our model, further quantitative analysis would be required to provide a more definitive evaluation of the model’s performance. Additionally, the work of Mincholé et al. (15) mainly focused on the QRS complex of the ECG, while the other crucial components of the ECG waveform, such as the P and T waves, have not been examined. To address this limitation, our future work will focus on integrating detailed biophysical parameters into our generative model, enabling a more precise quantitative comparison between our synthesized ECGs and simulation-based results.

About the CVD risk prediction model, the results from Figure 5 suggest that our cVAE with predictor successfully learned to stratify subjects by CVD risk using features extracted from 12-lead ECG signals. This indicates that there is useful information related to their future CVD risk contained in ECG recordings, and our model has the ability to capture it. The baseline model in the current study attained a C-index of 0.63, suggesting a moderate predictive performance that necessitates further refinement. Although the C-index provides a useful quantification of model performance, its standalone value might not fully encapsulate the model’s clinical applicability. The future works could further explore the ECG of the subjects defined as high risk group by our network, and analyze their ECG measurements in detail in order to find common characteristics for certain diseases.

When we include additional demographic information to our prediction network, as shown in Tables 1 and 2, it improves the C-index by 3% and 5%. This is consistent with previous findings of Alaa et al. (20), which highlighted the importance of age and sex in CVD risk evaluation. While this suggests that sex and age contribute to risk prediction, the relatively modest increase reflects the fact that ECG waveforms already encode physiological characteristics associated with these demographic factors. Our future work will explore the inclusion of additional subject information commonly used in traditional risk evaluation methods, such as the Framingham Risk Score factors (e.g., smoking history, blood pressure), to assess whether incorporating a broader range of clinical variables could further enhance model performance.

In Table 2, we notice that the addition of electrodes’ positions does not improve the C-index. One possible explanation is that the relationship between electrodes’ positions and CVD risk is already partially captured within the ECG waveforms themselves. Since ECG morphology inherently encodes subject-specific anatomical and physiological characteristics, some of the variability introduced by differences in electrode positioning may have already been learned by the model. Due to the high dimensionality of the electrode position data (3×10 coordinates), the single fully connected layer in our current model may not be expressive enough to fully map these features into the latent space for risk prediction. A more complex network architecture could be explored in future to better leverage electrode position information for improved prediction performance.

While our study investigates general CVD risk prediction, further work is needed to explore how changes in ECG amplitude and duration, resulting from variations in heart position and electrode placement, impact the prediction of specific cardiovascular diseases. Certain ECG-derived biomarkers, such as ST-segment deviations or QRS complex amplitudes, are directly influenced by these factors and play a crucial role in diagnosing conditions such as myocardial infarction or hypertrophy. A future extension of our work could involve evaluating how disease-specific classification models respond to these anatomical influences, improving the interpretability and robustness of ECG-based prediction methods.

## 5 Conclusion

In this work, we have developed a cVAE-based ECG generation model, incorporating the electrodes’ positions to include torso information. This approach has markedly improved the consistency between our generated signals and previous *in silico* studies, surpassing the performance of models that relied solely on heart position and orientation. Through the meaningful latent space representation learned by our cVAE model, we highlight the ability of ECG signals alone to predict future CVD risk. Furthermore, by incorporating additional conditioning factors such as age, sex, and electrodes’ positions, we demonstrate that these structured inputs provide additional guidance, further refining risk estimation. Our findings underscore the potential of generative approaches to extract clinically relevant features from 12-lead ECG signals, supporting the development of more personalized and data-driven CVD risk assessment models.

## Data Availability

The data supporting the conclusions of this article will be made available by the authors upon reasonable request.

## References

[1] Waller AD. A demonstration on man of electromotive changes accompanying the heart’s beat. J Physiol (Lond). (1887) 8:229–34. 10.1113/jphysiol.1887.sp000257 PMC1485094 16991463

[2] George S Rodriguez I Ipe D Sager PT Gussak I Vajdic B. Computerized extraction of electrocardiograms from continuous 12-lead holter recordings reduces measurement variability in a thorough QT study. J Clin Pharmacol. (2012) 52:1891–900. 10.1177/0091270011430505 22187440

[3] Beetz M Banerjee A Grau V. Multi-domain variational autoencoders for combined modeling of MRI-based biventricular anatomy and ECG-based cardiac electrophysiology. Front Physiol. (2022) 13:886723. 10.3389/fphys.2022.886723 35755443 PMC9213788

[4] Beetz M Banerjee A Sang Y Grau V. Combined generation of electrocardiogram and cardiac anatomy models using multi-modal variational autoencoders. In: *2022 IEEE 19th International Symposium on Biomedical Imaging (ISBI)* (2022). p. 1–4.

[5] Benali R Bereksi Reguig F Hadj Slimane Z. Automatic classification of heartbeats using wavelet neural network. J Med Syst. (2012) 36:883–92. 10.1007/s10916-010-9551-7 20703646

[6] Kampouraki A Manis G Nikou C. Heartbeat time series classification with support vector machines. IEEE Trans Inf Technol Biomed. (2008) 13:512–8. 10.1109/TITB.2008.2003323 19273030

[7] Li L Camps J Banerjee A Beetz M Rodriguez B Grau V. Deep computational model for the inference of ventricular activation properties. In: *Statistical Atlases and Computational Models of the Heart. Regular and CMRxMotion Challenge Papers: 13th International Workshop, STACOM 2022, Held in Conjunction with MICCAI 2022, Singapore, September 18, 2022, Revised Selected Papers*. Springer (2023). p. 369–80.

[8] Li L Camps J Jenny Wang Z Beetz M Banerjee A Rodriguez B, et al. Toward enabling cardiac digital twins of myocardial infarction using deep computational models for inverse inference. IEEE Trans Med Imaging. (2024) 43:2466–78. 10.1109/TMI.2024.3367409 38373128 PMC7616288

[9] Zhang L Peng H Yu C. An approach for ECG classification based on wavelet feature extraction and decision tree. In: *2010 International Conference on Wireless Communications & Signal Processing (WCSP)*. IEEE (2010). p. 1–4.

[10] Chen C Li L Beetz M Banerjee A Gupta R Grau V. Large language model-informed ECG dual attention network for heart failure risk prediction. *arXiv* [Preprint]. *arXiv:2403.10581* (2024). 10.1109/TBDATA.2025.3536922 PMC7617765 40524840

[11] McLachlan S Dube K Gallagher T. Using the caremap with health incidents statistics for generating the realistic synthetic electronic healthcare record. In: *2016 IEEE International Conference on Healthcare Informatics (ICHI)*. IEEE (2016). p. 439–48.

[12] Esteban C Hyland SL Rätsch G. Real-valued (medical) time series generation with recurrent conditional gans. *arXiv* [Preprint]. *arXiv:1706.02633* (2017).

[13] Kuznetsov V Moskalenko V Zolotykh NY. Electrocardiogram generation and feature extraction using a variational autoencoder. *arXiv* [Preprint]. *arXiv:2002.00254* (2020).

[14] Zhu F Ye F Fu Y Liu Q Shen B. Electrocardiogram generation with a bidirectional LSTM-CNN generative adversarial network. Sci Rep. (2019) 9:6734. 10.1038/s41598-019-42516-z 31043666 PMC6494992

[15] Mincholé A Zacur E Ariga R Grau V Rodriguez B. MRI-based computational torso/biventricular multiscale models to investigate the impact of anatomical variability on the ECG QRS complex. Front Physiol. (2019) 10:458916. 10.3389/fphys.2019.01103 PMC6718559 31507458

[16] Jæger KH Tveito A. Deriving the bidomain model of cardiac electrophysiology from a cell-based model; properties and comparisons. Front Physiol. (2022) 12:811029. 10.3389/fphys.2021.811029 35069265 PMC8782150

[17] Sang Y Beetz M Grau V. Generation of 12-lead electrocardiogram with subject-specific, image-derived characteristics using a conditional variational autoencoder. In: *2022 IEEE 19th International Symposium on Biomedical Imaging (ISBI)*. IEEE (2022). p. 1–5.

[18] Smith HJ Banerjee A Choudhury RP Grau V. Automated torso contour extraction from clinical cardiac MR slices for 3D torso reconstruction. In: *2022 44th Annual International Conference of the IEEE Engineering in Medicine & Biology Society (EMBC)*. IEEE (2022). p. 3809–13. 10.1109/EMBC48229.2022.9871643 36086129

[19] Smith HJ Rodriguez B Sang Y Beetz M Choudhury R Grau V, et al. Anatomical basis of sex differences in human post-myocardial infarction ECG phenotypes identified by novel automated torso-cardiac 3D reconstruction. *arXiv* [Preprint]. *arXiv:2312.13976* (2023).

[20] Alaa AM Bolton T Di Angelantonio E Rudd JH Van der Schaar M. Cardiovascular disease risk prediction using automated machine learning: a prospective study of 423,604 UK Biobank participants. PLoS ONE. (2019) 14:e0213653. 10.1371/journal.pone.0213653 31091238 PMC6519796

[21] Emdin CA Wong CX Hsiao AJ Altman DG Peters SA Woodward M, et al. Atrial fibrillation as risk factor for cardiovascular disease and death in women compared with men: systematic review and meta-analysis of cohort studies. BMJ. (2016) 352:h7013. 10.1136/bmj.h7013 PMC5482349 26786546

[22] Turnbull I Camm C Halsey J Du H Chen Z Clarke R. Population prevalence of ECG abnormalities and risk of incident CVD outcomes: 5-year follow-up of 25,000 Chinese adults. Eur Heart J. (2023) 44:ehad655–2380. 10.1093/eurheartj/ehad655.2380

[23] Wu G Wu J Lu Q Cheng Y Mei W. Association between cardiovascular risk factors and atrial fibrillation. Front Cardiovasc Med. (2023) 10:1110424. 10.3389/fcvm.2023.1110424 37753167 PMC10518410

[24] Sudlow C Gallacher J Allen N Beral V Burton P Danesh J, et al. UK Biobank: an open access resource for identifying the causes of a wide range of complex diseases of middle and old age. PLoS Med. (2015) 12:e1001779. 10.1371/journal.pmed.1001779 25826379 PMC4380465

[25] Natarajan A Chang Y Mariani S Rahman A Boverman G Vij S, et al. A wide and deep transformer neural network for 12-lead ECG classification. In: *2020 Computing in Cardiology*. IEEE (2020). p. 1–4.

[26] Petersen SE Matthews PM Francis JM Robson MD Zemrak F Boubertakh R, et al. UK Biobank’s cardiovascular magnetic resonance protocol. J Cardiovasc Magn Reson. (2015) 18:1–7. 10.1186/s12968-016-0227-4 PMC4736703 26830817

[27] Strain T Wijndaele K Sharp SJ Dempsey PC Wareham N Brage S. Impact of follow-up time and analytical approaches to account for reverse causality on the association between physical activity and health outcomes in UK Biobank. Int J Epidemiol. (2020) 49:162–72. 10.1093/ije/dyz212 31651957 PMC7124507

[28] Banerjee A Camps J Zacur E Andrews CM Rudy Y Choudhury RP, et al. A completely automated pipeline for 3D reconstruction of human heart from 2D cine magnetic resonance slices. Philos Trans R Soc A. (2021) 379:20200257. 10.1098/rsta.2020.0257 PMC8543046 34689630

[29] Kingma DP Ba J. Adam: a method for stochastic optimization. *arXiv* [Preprint]. *arXiv:1412.6980* (2014).

[30] Harrell Jr FE Lee KL Califf RM Pryor DB Rosati RA. Regression modelling strategies for improved prognostic prediction. Stat Med. (1984) 3:143–52. 10.1002/sim.4780030207 6463451

[31] Pencina MJ D’agostino RB. Overall c as a measure of discrimination in survival analysis: model specific population value and confidence interval estimation. Stat Med. (2004) 23:2109–23. 10.1002/sim.1802 15211606

